# Exploring agile leadership, ambidextrous leadership, organizational agility, and innovation behaviors among nurse managers: an explanatory mixed-methods study

**DOI:** 10.3389/frhs.2026.1950319

**Published:** 2026-10-01

**Authors:** Ebtsam Aly Abou Hashish, Hend Alnajjar

**Affiliations:** 1 College of Nursing - Jeddah, King Saud bin Abdul-Aziz University for Health Sciences, Jeddah, Saudi Arabia; 2 King Abdullah International Medical Research Center, Jeddah, Saudi Arabia; 3 Ministry of National Guard Health Affairs, Jeddah, Saudi Arabia; 4 Faculty of Nursing, Alexandria University, Alexandria, Egypt

**Keywords:** agile leadership, ambidextrous leadership, innovation behaviors, mixed methods, nurse managers, organizational agility

## Abstract

**Aim:**

To describe agile leadership, ambidextrous leadership, organizational agility, and innovation behaviors among nurse managers and explore how these practices are experienced in clinical management.

**Background:**

Agile leadership, organizational responsiveness, and innovation are increasingly relevant to nursing management. However, these concepts may overlap substantially when measured through same-source self-report, making qualitative contextualization important for understanding how they are experienced in practice.

**Methods:**

An exploratory sequential explanatory mixed-methods study was conducted in a tertiary Saudi hospital. Forty-four nurse managers completed measures of agile leadership, ambidextrous leadership, organizational agility, and innovation behaviors. Thirty-two nurse managers then participated in semi-structured interviews analyzed thematically. Quantitative data were analyzed descriptively and exploratorily, and qualitative data were analyzed thematically. Greater interpretive weight was given to the qualitative phase.

**Results:**

Nurse managers reported moderate agile and ambidextrous leadership and high organizational agility and innovation behaviors. The four measures showed near-unity correlations, limiting construct-specific interpretation. Qualitative analysis generated five themes: agile leadership as flexibility and human-centered responsiveness; ambidextrous leadership as balancing opening and closing behaviors; organizational agility as adaptive unit functioning; innovation as problem-driven practice; and contextual challenges influencing leadership effectiveness.

**Conclusion:**

The findings indicate a highly interrelated agile leadership–organizational functioning profile. The primary contribution lies in the qualitative findings, which show how nurse managers described flexibility, delegation, balancing operational and improvement work, problem-driven innovation, and contextual constraints within Saudi clinical management. Larger multisite and multi-source studies are needed to determine whether the measured constructs are empirically distinct.

## Introduction

The rapidly evolving healthcare landscape in Saudi Arabia, driven by initiatives like Vision 2030, necessitates the adoption of innovative leadership strategies (1, 2). Nurse managers, central to the operational and strategic frameworks of healthcare organizations, are pivotal in balancing patient care excellence with organizational adaptability (3, 4). Leadership models such as agile and ambidextrous leadership have emerged as promising frameworks to address the dynamic challenges of modern healthcare environments (5). Agile leadership emphasizes adaptability, team collaboration, and proactive change management, enabling leaders to respond swiftly to unforeseen challenges (6).

Ambidextrous leadership complements agile leadership by describing how leaders balance exploration and exploitation through opening and closing behaviors (7, 8). Although agile leadership, ambidextrous leadership, organizational agility, and innovation behaviors are theoretically distinguishable, their behavioral content can converge around responsiveness, flexibility, empowerment, and improvement. Evidence from healthcare and the Middle East remains limited regarding how nurse managers experience these concepts together in practice (5, 9). This study therefore examines them as theoretically informed but potentially overlapping features of an agile leadership–organizational functioning profile, with qualitative inquiry providing the principal contextual interpretation of the quantitative pattern.

### Background and conceptual framework

This study was informed by the Agile Leadership Framework and Ambidexterity Leadership for Innovation (7, 10). These frameworks provided sensitizing concepts for examining agile and ambidextrous leadership practices rather than a basis for confirmatory testing of directional pathways. The four study concepts were retained because they originate from different theoretical literatures and instruments. Their empirical separability, however, could not be assumed in the present small, same-source dataset.

*Agile leadership* is a dynamic approach that emphasizes adaptability, continuous learning, and collaborative decision-making in complex and rapidly changing environments (5, 8). Grounded in the Agile Leadership Framework, it centers on responsiveness and flexibility as core leadership capabilities (10). Agile leaders empower teams, promote collaboration, and respond rapidly to evolving demands (6). This approach aligns closely with the realities of healthcare, where nurse managers navigate workforce diversity, technological change, and regulatory demands (6, 9). Within this context, agile leadership has been associated with continuous learning, innovation, proactive problem-solving, and adaptive team functioning. It also emphasizes decentralized decision-making and teamwork, which are essential in fast-paced clinical environments (10, 11).

Özgenel and Yazıcı (12) identified three core domains of agile leadership: situational awareness, human relations, and self-awareness. Situational awareness refers to a leader's capacity to read and interpret the environment accurately, anticipating shifts and responding proactively before challenges escalate (12). Human relations encompass the leader's ability to build trust, nurture meaningful interpersonal connections, and promote a culture of open communication and psychological safety within the team (11, 12). Self-awareness reflects the leader's ongoing reflection on their own values, strengths, and limitations, enabling authentic and consistent leadership behavior (12). Together, these three domains provide a practical foundation for agile leadership, guiding nurse managers to lead with clarity, empathy, and responsiveness in complex clinical settings (6, 9). Translating these domains into nursing practice, maintaining productive outcomes for nurses requires supportive leadership that empowers them and promotes positive work behaviors (13).

*Ambidextrous leadership* provides a dual framework for managing the competing demands of exploration (innovative thinking) and exploitation (efficiency) (7). This leadership model explains how leaders alternate between two behavioral domains: opening behaviors, which encourage creativity and experimentation, and closing behaviors, which focus on refinement and implementation (7, 14). Opening behaviors include practices such as granting autonomy, welcoming divergent ideas, and tolerating experimentation, while closing behaviors involve setting clear goals, monitoring progress, and reinforcing established routines to ensure operational consistency (7, 15). This duality is essential in healthcare, where operational excellence and innovation must coexist (5, 8). Previous research has associated ambidextrous leadership with organizational performance creating an environment where creativity thrives alongside operational excellence (8). Findings further suggest that ambidextrous leadership has been associated with cultures supporting continuous learning and innovation, enabling teams to adapt to technological advancements and evolving patient needs (15, 16); recent evidence also links ambidextrous leadership to lower nurse presenteeism, underscoring its relevance to staff wellbeing outcomes (17).

Agile and ambidextrous leadership remain conceptually distinguishable in the literature. Agile leadership emphasizes environmental awareness, relational responsiveness, flexibility, and self-reflection (12), whereas ambidextrous leadership focuses on the situational use of opening and closing behaviors to balance exploration and exploitation (7). In practice, both may be experienced by nurse managers as parts of a broader agile leadership repertoire. The present study therefore does not assume that these constructs can be empirically separated in this sample. Their measured overlap is interpreted alongside participants’ accounts.

*Organizational agility,* defined as the ability to adapt quickly to changing conditions, is a vital component of innovation in healthcare (18). Agile organizations are characterized by their ability to reconfigure processes, align resources, and foster collaboration — all of which are essential for addressing the complexities of patient care (6, 18). Sharifi and Zhang (18) described four dimensions of organizational agility: responsiveness, which refers to the capacity to sense and react to changes in a timely manner; flexibility, which reflects the ability to adjust processes, structures, and strategies as needed; competency, which captures the skills, knowledge, and capabilities required to act effectively under conditions of uncertainty; and quickness, which denotes the speed with which an organization can execute decisions and implement changes. Studies have reported associations between leadership and organizational agility, and leadership style has been identified as a correlate of organizational adaptability and innovation (8, 19).

*Nursing innovation* is a dynamic and multifaceted concept integral to enhancing the quality of healthcare delivery (20). Innovative behavior is a cornerstone of nursing innovation, a motivational and cognitive process characterized by activities that create, develop, and implement new ideas within healthcare settings (21). The International Council of Nurses highlights innovation as a pivotal mechanism for advancing health promotion, disease prevention, and patient care quality through novel practices and technologies (22).

Lukes and Stephan (21) identified six aspects of innovative behavior: idea generation, which involves recognizing opportunities and producing novel solutions; idea search, which refers to actively seeking information and knowledge to refine emerging ideas; idea communication, which encompasses sharing and advocating for new ideas among colleagues and stakeholders; implementation-starting activities, which involve initiating concrete steps to put ideas into action; involving others, which reflects the process of building coalitions and enlisting support for innovation efforts; and overcoming obstacles, which captures the persistence required to navigate organizational, resource-related, or interpersonal barriers to implementation (21). These dimensions aim to achieve tangible innovation outputs, such as the development of new products or the modification of existing services (21, 22). Studies reveal that innovative behaviors have been associated with innovation outputs, though this relationship is often mediated by contextual factors such as managerial and organizational support (22).

In the healthcare context, innovative behaviors have been linked to improved medical treatment effectiveness, care quality, and operational efficiency (20). Nurses’ innovative behaviors may contribute to these outcomes; however, other research indicates that innovative behaviors alone may not directly influence patient satisfaction or care quality, suggesting the need for supportive environments to realize the full potential of innovation (22). Organizational and managerial support are particularly influential, as they provide the necessary resources and a conducive environment for innovation to thrive (20, 22).

Research reported that agile and ambidextrous leaders create environments where employees feel empowered, fostering trust and collaboration (8, 11). Cross-sector public-sector evidence similarly suggests that leadership-related organizational conditions may shape discretionary employee behavior through broader institutional and ethical processes (23), reinforcing the need to consider the institutional context surrounding individual leadership behaviors. Such work environments have been associated with organizational adaptability and innovation-related outcomes (5, 6). In Saudi hospitals, where nurse managers face unique cultural and operational challenges, effective leadership is critical for promoting agility and improving organizational outcomes (1, 19).

Taken together, the four study concepts may reflect a broader agile leadership–organizational functioning profile in which responsiveness, flexibility, empowerment, and improvement are closely connected. The quantitative phase described the levels and covariation of the measures. The qualitative phase examined how nurse managers understood and enacted these practices and the contextual conditions surrounding them.

### Significance of the study

Effective leadership requires adaptability and the capacity to respond to changing conditions while learning from experience (24). In healthcare, this need is amplified by continuous shifts in technology, workforce composition, and regulatory demands. Within such environments, agile and ambidextrous leadership approaches have received increasing attention in relation to organizational performance and resilience (25). These leadership styles promote empowerment, collaboration, and responsiveness, enabling teams to function effectively under uncertainty (11, 24).

In Saudi hospitals, where nurses operate under high workloads within culturally diverse teams, empowering leadership is particularly important for enhancing work outcomes and maintaining care quality (26). Despite this relevance, existing research on agile and ambidextrous leadership has largely focused on industrial contexts, with limited application to healthcare settings (5, 10). While prior studies have highlighted the role of agile leadership in fostering adaptability (6) and ambidextrous leadership in balancing innovation and efficiency (7, 15), their implications within healthcare systems—especially in Saudi Arabia—remain insufficiently examined.

Furthermore, although innovative behaviors are recognized as essential for healthcare improvement (20, 22), the leadership-related organizational factors associated with these behaviors are not well established. In particular, empirical evidence on the possible statistical relationship of organizational agility with leadership and innovation behaviors remains limited (5, 8).

This study addresses these gaps by combining a descriptive quantitative profile with in-depth qualitative accounts of agile and ambidextrous leadership, organizational agility, and innovation among Saudi nurse managers. Rather than treating the four measures as empirically distinct predictors, the study uses their observed covariation as contextual evidence and positions participants’ accounts as the main source of insight into how these practices are experienced in the clinical setting.

### Aim of the study

This study aimed to describe the profile and covariation of agile leadership, ambidextrous leadership, organizational agility, and innovation behaviors among nurse managers, and to explore qualitatively how nurse managers experienced these leadership practices, organizational agility, innovation, and contextual constraints. The qualitative phase provided the primary interpretive contribution, while the quantitative findings served as descriptive and exploratory context.

### Research questions

RQ1. What descriptive levels and patterns of covariation are observed across agile leadership, ambidextrous leadership, organizational agility, and innovation behaviors among nurse managers?.

RQ2. How do nurse managers describe agile and ambidextrous leadership practices, balancing operational and improvement demands, organizational agility, and innovation in everyday clinical management?.

RQ3. What contextual and organizational conditions shape these leadership and improvement practices in the study setting?.

RQ4. How do the qualitative findings contextualize the highly interrelated quantitative profile observed across the four measures?.

## Methods

### Study design and setting

This study employed a sequential explanatory mixed-methods design (27), with a quantitative survey phase followed by a qualitative interview phase. The quantitative phase described levels and covariation across four theoretically informed measures. Regression analyses were retained only as exploratory diagnostics of coefficient behavior under substantial construct overlap. The qualitative phase then explored how nurse managers described agile and ambidextrous leadership practices, organizational agility, innovation, and contextual constraints. In the integrated interpretation, greater weight was assigned to the qualitative findings because the quantitative data could not establish empirical separability among the four constructs (Figure 1).

**Figure 1:**
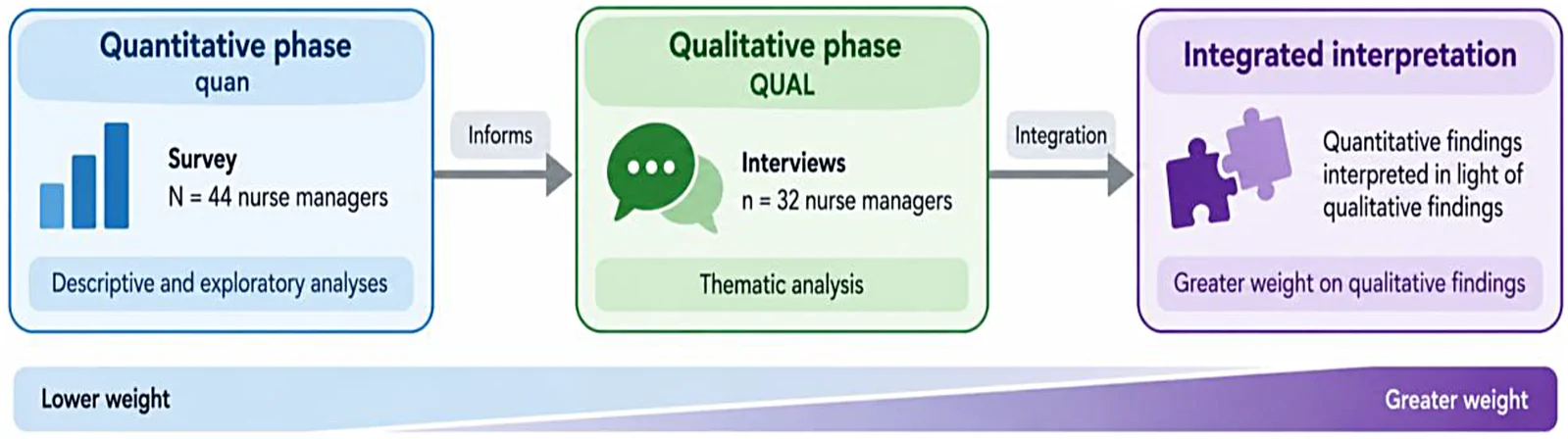
Sequential explanatory mixed-methods design and interpretive weighting.

The study was conducted in inpatient care units at King Khaled Hospital (KKH). KKH is a 750-bed tertiary care facility accredited by the Joint Commission International and characterized by complex clinical operations. Its multinational nursing workforce, diverse clinical departments, and dual operational and innovation imperatives made it an ideal setting for examining the relationships of interest.

### Participants and sampling

#### Quantitative phase

The target population comprised all nurse managers working in inpatient care units at KKH (*N* = 50). A census approach was applied, and all eligible nurse managers were invited. A total of 44 nurse managers agreed to participate. Because all eligible nurse managers were invited, the quantitative phase represented a census of the available organizational population rather than a sample derived from an *a priori* power calculation. Inclusion criteria required a minimum of six months’ experience in the current managerial role and availability during the data collection period. Bedside nurses and non-managerial staff were excluded.

#### Qualitative phase

A purposive sample of 32 nurse managers was drawn from the quantitative participants to ensure variation in clinical specialty, years of experience, and nationality. Sampling continued until no new codes or substantive insights were identified in two consecutive interviews, with subsequent interviews confirming the stability of the thematic structure. Because interview identifiers were stored separately from the quantitative analytic file to preserve confidentiality, participant-level linkage was unavailable at the analysis stage. A direct quantitative comparison between interviewed and non-interviewed participants could therefore not be conducted. Purposive selection was based on clinical specialty, managerial experience, and nationality to maximize variation.

### Data Collection Instrument

Quantitative data were collected using a validated self-administered questionnaire composed of five sections:

  Section 1: Demographic and professional characteristics. Participants provided information on gender, age, nationality, educational level, clinical work area, years of managerial experience, average weekly hours, and whether they worked extra shifts during the month.

  Section 2: Agile Leadership Scale. Agile leadership was measured using the Marmara Agile Leadership Scale developed by Özgenel and Yazıcı (12). The scale comprises 34 items across three subscales: situational awareness (12 items), human relations (14 items), and self-awareness (8 items). Items were rated on a five-point Likert scale from 0 (Never) to 4 (Always), with higher scores indicating stronger agile leadership characteristics. Mean scores are interpreted as follows: 0.00–0.79 = very low, 0.80–1.59 = low, 1.60–2.39 = medium, 2.40–3.19 = high, and 3.20–4.00 = very high. The original scale demonstrated excellent reliability (Cronbach's *α* = 0.98), confirmed in the present sample (*α* = 0.98).

  Section 3: Ambidextrous Leadership Scale. Ambidextrous leadership was assessed using the Zacher and Rosing (28) scale, grounded in Rosing et al.'s (7) theoretical framework. The scale contains 14 items: seven assessing opening leader behaviors and seven assessing closing leader behaviors. Items were rated on a five-point Likert scale from 0 (Never) to 4 (Always). The scale has demonstrated strong reliability in healthcare settings [Cronbach's *α* = 0.90 (8);] and yielded *α* = 0.93 in the current study.

  Section 4: Organizational Agility Scale. Organizational agility was measured using the scale developed by Sharifi and Zhang (18), comprising 17 items across four dimensions: responsiveness (3 items), flexibility (3 items), competency (8 items), and quickness (3 items). Items were rated on a five-point Likert scale from 1 (Strongly Disagree) to 5 (Strongly Agree). The scale has demonstrated strong reliability in organizational settings including healthcare [*α* = 0.87 (19);], and yielded *α* = 0.98 in the present sample.

  Section 5: Innovative Behavior Inventory (IBI). Innovation behaviors were assessed using the IBI developed by Lukes and Stephan (21), comprising 23 items across seven subscales: idea generation (3 items), idea search (3 items), idea communication (4 items), implementation starting (3 items), involving others (3 items), overcoming obstacles (4 items), and innovation outputs (3 items). Items were rated on a five-point Likert scale from 1 (Strongly Disagree) to 5 (Strongly Agree). The IBI has demonstrated cross-cultural reliability [Cronbach's *α* ranging from 0.60 to 0.88 across subscales (21);] and yielded total *α* = 0.97 in the present study.

For qualitative data Semi-structured interviews were conducted to explore nurse managers’ perspectives and experiences of agile and ambidextrous leadership and their perceived relationships with organizational agility and innovation behaviors. The interview guide developed by the researchers included two parts. The first part captured background information such as years of leadership experience and working area. The second part included open-ended questions where participants were asked to describe how they apply agile leadership in daily work, how leadership practices relate to team innovation from participants’ perspectives, and what challenges they face in managing competing demands. Interviews were conducted individually, allowing participants to share detailed accounts of their experiences. Data collection continued until data saturation was achieved.

### Validity and reliability

All instruments were previously validated in their original contexts. Prior to data collection, the questionnaire was reviewed by an expert panel of five academic and clinical nursing specialists to assess content validity and suitability for the Saudi healthcare setting. Minor linguistic refinements were made to ensure clarity and contextual relevance.

A pilot study was conducted with four participants to evaluate clarity, comprehension, and feasibility of the response format. No major modifications were required. Reliability was assessed using Cronbach's alpha, with a threshold of *α* ≥ 0.70 indicating acceptable internal consistency (29). These coefficients indicate internal consistency only and do not establish construct distinctiveness. Although internal consistency coefficients were high, values approaching 0.97–.98 may also reflect item redundancy and should not be interpreted as evidence of discriminant validity. For the qualitative phase, the interview guide was developed based on the study variables and research objectives, then reviewed by the same expert panel to ensure content relevance and clarity. Two pilot interviews were conducted to assess question flow, clarity, and probing adequacy. Minor refinements were made to wording and sequencing before final data collection.

### Data collection

Approval was obtained from the King Abdullah International Medical Research Center (KAIMRC) IRB and KKH hospital director prior to data collection. Eligible nurse managers were approached individually by the research team, provided with a participant information sheet explaining the study purpose and procedures, and invited to participate voluntarily. Written informed consent was obtained before data collection, and participants were informed of their right to withdraw at any time without consequence.

Quantitative data were collected using structured questionnaires distributed both in person and electronically via institutional email. Participants were given a two-week period to complete and return the questionnaires.

Qualitative data were collected through semi-structured interviews conducted in nurse managers’ offices, scheduled during or adjacent to working hours based on participants’ availability. The interviews followed a predefined guide while allowing participants to share their experiences in depth. Interviews lasted approximately 30–45 min and were conducted by the researchers. With participants’ permission, interviews were audio-recorded and transcribed verbatim. All interviews were conducted in English, and no translation was required. Data collection took place between September and November 2025.

### Data analysis

Quantitative data were coded and analyzed using the Statistical Package for the Social Sciences (SPSS), version 28. Descriptive statistics, including frequencies, percentages, means, and standard deviations, were used to summarize demographic characteristics and study variables. Pearson correlation analysis was conducted to examine bivariate associations among the study variables.

Pearson correlations were used to describe the degree of covariation among the four measures. Exploratory multiple regression models were retained as descriptive sensitivity analyses to show how coefficients behaved when the two leadership measures were entered simultaneously. Because the constructs were near-unity correlated, the regression coefficients were not interpreted as estimates of unique effects or as tests of theoretically distinct predictors. Variance inflation factors (VIFs) were examined to document multicollinearity. Harman's single-factor test was used only as a limited diagnostic of potential common-method variance because all quantitative measures were self-reported by the same respondents at the same time (30).

No mediation or indirect-association model was retained in the reframed analysis. Given the cross-sectional design, same-source measurement, near-unity correlations, severe multicollinearity, and inability to establish discriminant validity, decomposing associations into direct and indirect components would imply a level of construct differentiation that the present data cannot support. Statistical significance was set at *p* ≤ 0.05 for descriptive inferential output, but emphasis was placed on the magnitude and pattern of covariation and on the limitations affecting interpretation.

Confirmatory factor analysis was not conducted because the 88 items across the four multi-item scales were measured in 44 participants, resulting in an item-to-case ratio below commonly recommended levels for stable factor estimation (31). Consequently, the present analysis could not establish discriminant validity among the four constructs.

A *post hoc* sensitivity analysis was conducted using GPower version 3.1.9.7 to determine the minimum effect size detectable with the available quantitative sample. With *N* = 44, *α* = 0.05, and 80% power, the minimum detectable effect size was approximately f^2^ = 0.24 for a multiple regression model with two predictors and f^2^ = 0.27 for a three-predictor model. These estimates were used to characterize the sensitivity of the available census sample rather than to establish adequacy for confirmatory path analysis.

Qualitative data were analyzed using Braun and Clarke’s (32) six-phase thematic analysis approach. The analysis began with familiarization through repeated reading of the transcripts and note-taking to capture initial impressions. Initial codes were then generated systematically across the dataset and grouped into potential themes reflecting patterns in participants’ experiences. The themes were reviewed against the coded extracts and the full dataset, refined, and clearly defined and named. The final themes were integrated into a coherent narrative supported by representative participant quotations and interpreted in relation to the study framework.

A hybrid inductive–deductive approach was applied. Initial coding was grounded in participants’ language and experiences, while subsequent organization and interpretation considered the study concepts of agile leadership, ambidextrous leadership, organizational agility, and innovation behaviors. Codes that did not align with these predefined concepts were retained when they represented relevant patterns emerging from the data.

Two researchers independently coded selected transcripts to assess coding consistency. Coding discrepancies were discussed and resolved through consensus. Saturation was assessed concurrently with data collection by both researchers through ongoing review of emerging codes and themes. Data collection continued until no new codes or substantive insights emerged across successive interviews, with subsequent interviews confirming the stability of the thematic structure.

Member checking was conducted by sharing brief summaries of the preliminary themes and interpretations with selected participants to assess whether they reflected their experiences and perspectives. Participant feedback supported the interpretation of the findings and did not result in substantive changes to the final themes.

Reporting of the qualitative component was informed by the Consolidated Criteria for Reporting Qualitative Research (COREQ). Trustworthiness was addressed through credibility, dependability, confirmability, and transferability (33). Credibility was supported through member checking, independent coding, and consensus discussions. Dependability was supported through an audit trail and reflexive notes maintained throughout the analytic process. Confirmability was addressed through researcher discussion and consensus on coding decisions and theme definitions. Transferability was supported through detailed description of the study setting, participant characteristics, and organizational context. The qualitative data analysis followed a hybrid inductive–deductive process, progressing from transcript familiarization and initial coding through theme development, researcher consensus, and member checking. See Figure 2 for the qualitative data analysis process.

**Figure 2:**
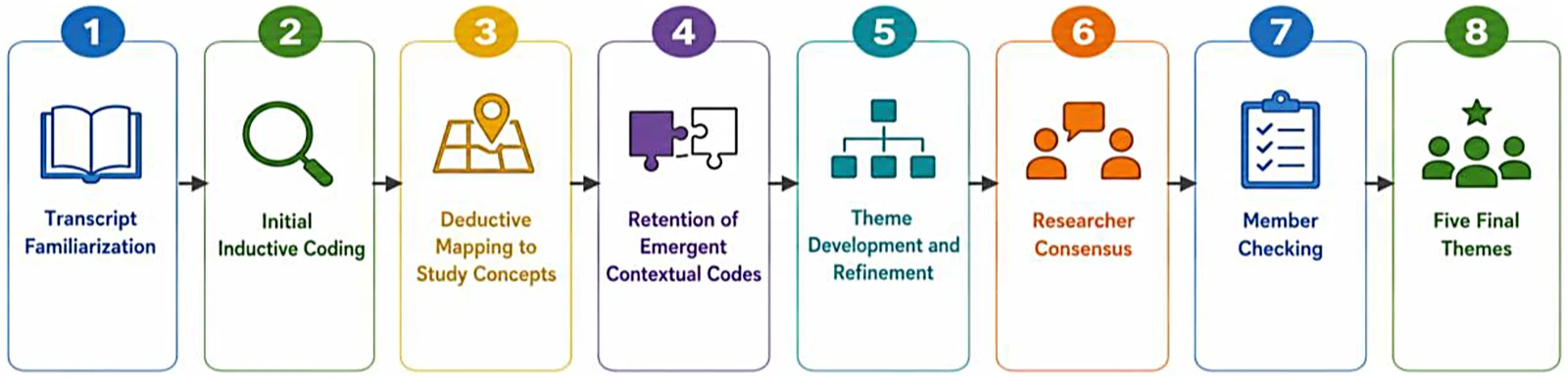
Qualitative data analysis approach.

### Ethical considerations

Ethical approval was obtained from KAIMRC prior to study commencement (IRB No. NRJ25/008/5). All participants provided written informed consent before participation. The research team explained the study purpose, procedures, and participants’ rights. Participation was voluntary, and participants could withdraw at any time without consequence. Anonymity was maintained by assigning alphanumeric codes (e.g., P1, P2) instead of names. No identifying information was reported, and all data were handled with strict confidentiality.

## Results

### Participant characteristics

The quantitative sample comprised 44 nurse managers, predominantly female (*n* = 40, 90.9%), with four male nurse managers also represented (*n* = 4, 9.1%). Participants were equally distributed between Saudi (50.0%) and non-Saudi nationalities (50.0%). The majority were aged 30 to <40 years (65.9%), and held a Bachelor's degree in nursing (70.5%). The intensive care unit was the most represented work area (20.5%), followed by surgical wards (18.2%). Most participants had 1–10 years of managerial experience (88.7%), and worked extra shifts during the month (79.5%). The mean working hours per week was 45.27 (SD = 2.41). Full demographic details are provided in Table 1.

**Table 1:** Demographic and professional characteristics of study participants (*N* = 44).

Characteristic	Category	N	%
Gender	Female	40	90.9
	Male	4	9.1
Nationality	Saudi	22	50.0
	Non-Saudi	22	50.0
Age	20–<30 years	6	13.6
	30–<40 years	29	65.9
	40–<50 years	9	20.5
Education	Bachelor's Degree	31	70.5
	Master's Degree	13	29.5
Work Area	Medical Ward	7	15.9
	Surgical Ward	8	18.2
	Obstetrics Ward	4	9.1
	Emergency Department	6	13.6
	Pediatric Ward	6	13.6
	Intensive Care Unit	9	20.5
	Oncology Ward	4	9.1
Years of Experience	1–5 years	20	45.5
	6–10 years	19	43.2
	11–15 years	5	11.4
Extra Shifts/Month	Yes	35	79.5
	No	9	20.5
Average Hours/Week	Mea*n* = 45.27, SD = 2.41		

### Descriptive statistics and internal consistency reliability

Descriptive statistics and Cronbach's alpha (*α*) are presented in Table 2. Agile leadership demonstrated a moderate level on the 0–4 scale (M = 2.90, SD = 0.49, *α* = 0.98). Among its subscales, self-awareness was highest (M = 2.93, SD = 0.51), followed by situational awareness (M = 2.90, SD = 0.50) and human relations (M = 2.89, SD = 0.48). Ambidextrous leadership was similarly moderate (M = 2.88, SD = 0.48, *α* = 0.93), with closing behaviors (M = 2.91) marginally higher than opening behaviors (M = 2.84). Innovation behaviors were rated high (M = 3.80, SD = 0.53, *α* = 0.97), with idea generation (M = 3.87) and idea search (M = 3.86) as the highest dimensions. Organizational agility was also high (M = 3.63, SD = 0.57, *α* = 0.97), with quickness leading (M = 3.78), followed by competency (M = 3.62), responsiveness (M = 3.61), and flexibility (M = 3.55). Figure 3 presents the distribution of the main study variable scores.

**Table 2:** Descriptive statistics and internal consistency reliability of study variables.

Scale/Subscale	Items	M	SD	Range	Cronbach's *α*
Agile Leadership total	34	2.90	0.49	2.24–3.82	0.975
Situational Awareness	12	2.90	0.50	2.25–3.83	0.929
Human Relations	14	2.89	0.48	2.21–3.79	0.932
Self-Awareness	8	2.93	0.51	2.25–3.88	0.897
Ambidextrous Leadership total	14	2.88	0.48	2.21–3.79	0.929
Opening Behaviors	7	2.84	0.52	2.14–3.86	0.873
Closing Behaviors	7	2.91	0.45	2.29–3.71	0.820
Innovation Behaviors total	23	3.80	0.53	3.00–4.87	0.970
Idea Generation	3	3.87	0.57	3.00–5.00	0.779
Idea Search	3	3.86	0.55	3.00–5.00	0.748
Idea Communication	4	3.81	0.49	3.00–4.75	0.721
Implementation Starting	3	3.79	0.58	3.00–5.00	0.767
Involving Others	3	3.80	0.55	3.00–5.00	0.706
Overcoming Obstacles	4	3.73	0.52	3.00–4.75	0.779
Innovation Outputs	3	3.77	0.58	3.00–5.00	0.760
Organizational Agility total	17	3.63	0.57	2.76–4.88	0.970
Competency	8	3.62	0.60	2.75–4.88	0.942
Flexibility	3	3.55	0.56	2.67–4.67	0.772
Responsiveness	3	3.61	0.58	2.67–5.00	0.805
Quickness	3	3.78	0.57	3.00–5.00	0.718

Agile Leadership and Ambidextrous Leadership scored on 0–4 scale. Innovation Behaviors and Organizational Agility scored on 1–5 Likert scale. All Cronbach's α ≥ 0.70.

**Figure 3:**
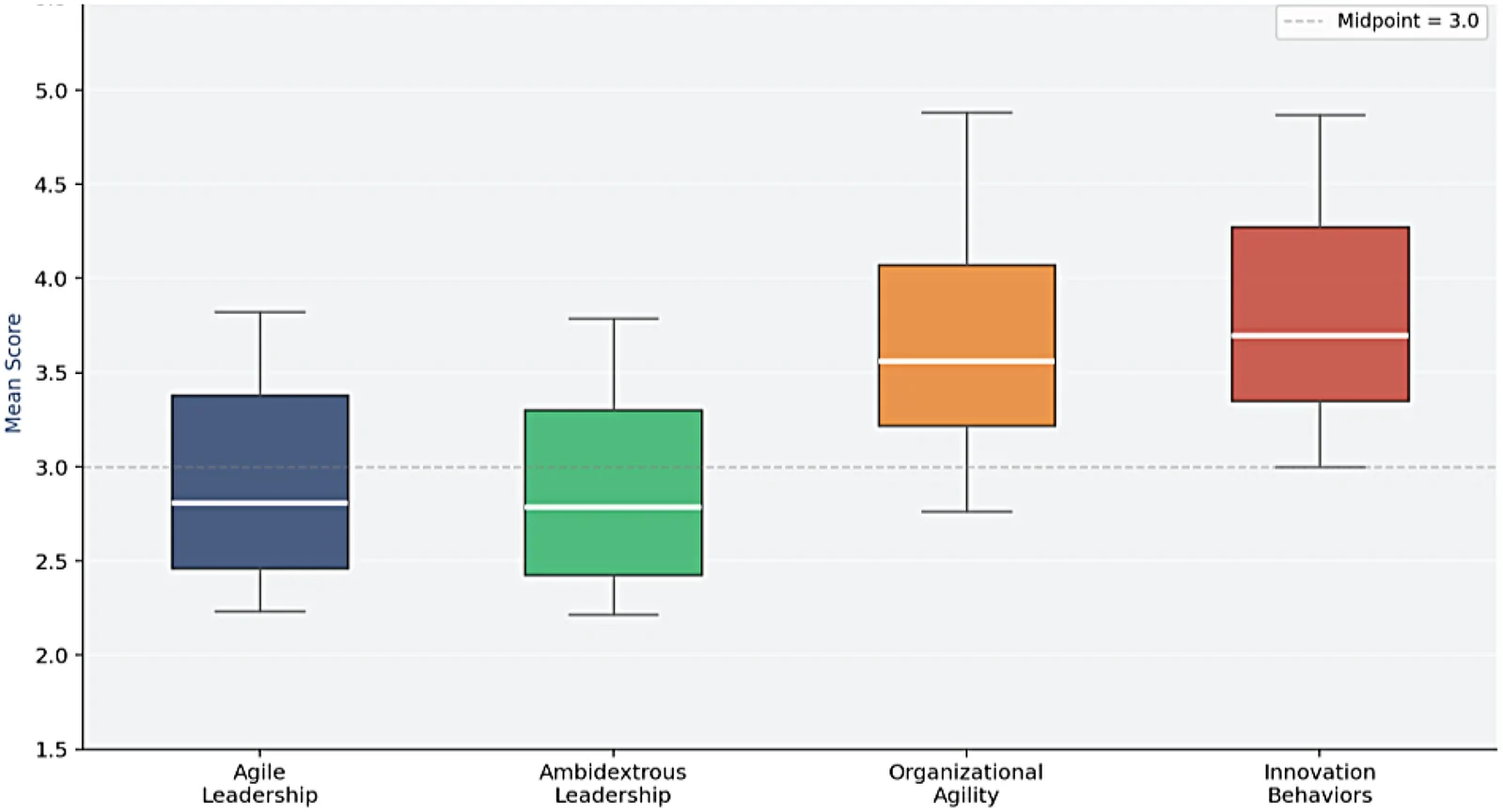
Distribution of main study variable scores.

### Exploratory quantitative profile and construct overlap

Pearson correlations showed near-unity positive covariation among all four measures (r = 0.977–.990, *p* < 0.001; Supplementary Table 1). This pattern indicates substantial empirical overlap among the measured domains. The quantitative results are therefore interpreted as a highly interrelated agile leadership–organizational functioning profile rather than as evidence of distinct construct-specific relationships.

The exploratory regression models are shown in Table 3 only to describe coefficient behavior under simultaneous entry of the two leadership measures. In the organizational agility model, the standardized coefficients were *β* = 0.77 for agile leadership and *β* = 0.21 for ambidextrous leadership. In the innovation-behavior model, they were *β* = 0.78 and *β* = 0.22, respectively. Model R^2^ values were 0.968 and 0.979. These values should not be read as evidence that agile leadership has a distinct or stronger effect. Rather, the extreme shared variance among predictors makes the conditional coefficients unstable and limits construct-specific interpretation.

**Table 3:** Exploratory descriptive regression models showing coefficient behavior under construct overlap.

Variable	OA B	SE	*β*	t	p	IB B	SE	β	t	p
Constant	0.29	0.09	—	3.03	.004	0.70	0.07	—	9.48	< 0.001
Agile leadership	0.90	0.20	.77	4.41	< 0.001	0.83	0.16	.78	5.19	< 0.001
Ambidextrous leadership	0.26	0.21	.21	1.23	.227	0.24	0.16	.22	1.47	.151

Model fit for OA: R^2^ = 0.968, adjusted R^2^ = 0.967, F(2, 41) = 622.90, *p* < 0.001.

Model fit for IB: R^2^ = 0.979, adjusted R^2^ = 0.978, F(2, 41) = 948.60, *p* < 0.001.

OA, organizational agility; IB, innovation behaviors; B, unstandardized coefficient; SE, standard error; β, standardized coefficient; VIF, 41.31 for the leadership predictors.

Multicollinearity diagnostics confirmed this limitation. VIF values reached 41.31 in the two-predictor models, far above conventional thresholds (31, 34). Harman's first unrotated factor accounted for 58.16% of total variance (30), which is consistent with substantial shared measurement influence but does not quantify common-method bias. Taken together, the correlations, VIFs, same-source design, and very high internal consistency coefficients prevent reliable estimation of the unique contribution of individual constructs in this dataset.

### Qualitative findings

Semi-structured interviews were conducted with 32 nurse managers across the hospital. Participants’ experience ranged from 2 to 14 years. Full qualitative sample characteristics are provided in Supplementary Table 2.

### Thematic findings

The qualitative findings constitute the primary interpretive contribution of the study. Five themes showed how nurse managers understood and enacted agile leadership, balancing behaviors, unit-level adaptation, problem-driven innovation, and contextual constraints. Rather than treating these themes as validation of four empirically distinct survey constructs, they are presented as participants’ accounts of an interconnected agile leadership–organizational functioning profile. See Table 4.

**Table 4:** Summary of qualitative themes and sub-themes .

Theme	Sub-themes
Theme 1 Agile Leadership as Flexibility and Human-Centered Responsiveness	1.1 Real-time adjustment to clinical demands
	1.2 Presence and floor visibility
	1.3 From reactive to anticipatory: experience gradient
	1.4 The human dimension of Leadership
Theme 2 Ambidextrous Leadership as Balancing Opening and Closing Behaviors	2.1 Unfamiliarity with the concept; intuitive practice
	2.2 Opening behaviors: creating space for new ideas
	2.3 Closing behaviors: sustaining change through monitoring
Theme 3 Organizational Agility as Adaptive Unit Functioning	3.1 Unit-level absorption of the unexpected
	3.2 Delegation in adaptive unit functioning
	3.3 Cultural and religious demands as recurring agility requirements
Theme 4 Innovation Behaviors as Problem-Driven Practices	4.1 Bedside observation triggers improvement
	4.2 Scale of innovation varies by seniority and role
	4.3 Working through staff resistance
Theme 5 Contextual Challenges Influencing Leadership Effectiveness	5.1 Physician hierarchy as a structural barrier
	5.2 Staff emotional state as prerequisite for innovation
	5.3 Time poverty under operational pressure
	5.4 Cultural dynamics within teams and with families

#### Theme 1: agile leadership as flexibility and human-centered responsiveness

Participants across all clinical settings described agile leadership as the capacity to adjust their approach based on what was unfolding around them in real time. These behaviors were consistent across specialties, though their pace and trigger varied: minute-to-minute in the ICU and Emergency Department, operating-list-driven in Surgical wards, and family-dynamic-responsive in Pediatrics and Obstetrics. Three subthemes captured this theme:

##### Subtheme 1.1: Real-time adjustment to clinical demands

Participants consistently described agile leadership in terms of daily, situation-driven adjustments to staffing, patient flow, and team assignments. Flexibility was not experienced as a planned strategy but as a continuous, responsive practice embedded in every shift.

“*Agile leadership for me means I must always be ready to adapt and change. Things change every day — patient condition, staffing, bed capacity. I cannot stay fixed in one plan. I observe the situation in the morning, I adjust my team assignment, and by afternoon I may change again.”*—Nurse Manager, Medical Ward, 5 years (P1)

##### Subtheme 1.2: Presence and Floor Visibility

Participants emphasized that agile leadership required physical presence on the ward floor rather than management from behind a desk. Being visible enabled early detection of emerging problems and faster intervention before situations escalated.

“*Agile leadership means you don't manage from your office — you are on the floor, you see what is happening, and you respond.”*—Nurse Manager, Pediatric Ward, 6 years (P22)

##### Subtheme 1.3: From Reactive to Anticipatory—An Experience Gradient

This qualitative pattern suggested differences in how managers at different experience levels described agile leadership. The cross-sectional quantitative analysis did not test developmental change over time.

“*After twelve years in nursing management, agility is not just reacting to crises. It is anticipating them and positioning your team to respond before the crisis fully develops.”*—Nurse Manager, Medical Ward, 12 years (P10)

##### Subtheme 1.4: The Human Dimension—Responding to People, Not Just Situations

Beyond clinical responsiveness, participants described agile leadership as attentiveness to the personal circumstances of both patients and staff. Flexibility in communication style, team management, and family engagement was seen as equally central to agility as operational adjustments.

“*Agile for me means I read the situation — the clinical situation and the family situation — and I adjust my approach accordingly. You have to be flexible with your team assignments but also with how you communicate and manage families.”*—Nurse Manager, Pediatric Ward, 6 years (P6)

#### Theme 2: ambidextrous leadership as balancing opening and closing behaviors

Ambidextrous leadership was unfamiliar as a formal concept to most participants, particularly those with Bachelor's-level education and fewer than six years of experience. When asked to describe specific situations, however, participants gave accounts that directly reflected opening and closing behavior dimensions (7). The gap between conceptual unfamiliarity and behavioral enactment was one of the most consistent findings across the dataset. Three subthemes emerged: unfamiliarity with the concept alongside familiarity with the practice, enactment of opening behaviors, and enactment of closing behaviors.

##### Subtheme 2.1: Unfamiliarity with the Concept, Familiarity with the Practice

Most participants had not encountered the term ambidextrous leadership in formal training. When the concept was explained, many recognized it as something they were already doing, though without a structured or intentional framework. Participants with Master's-level education and extended experience were notably more precise in their articulation.

“*Ambidextrous leadership is simultaneously excelling at both exploitation — using existing knowledge and systems to deliver today's results — and exploration — generating new knowledge and practices for tomorrow's improvement. A manager who only exploits will stagnate; a manager who only explores will neglect today's patients.”*—Nurse Manager, Medical Ward, 12 years (P9)

##### Subtheme 2.2: Opening Behaviors—Making Room for Change While the Ward Runs

Opening behaviors were described as creating space for new ideas, encouraging experimentation, and initiating improvement work in parallel with ongoing operational responsibilities. Participants enacted these behaviors informally during shifts rather than in dedicated improvement time.

“*I noticed our central line care bundle compliance was low. During my shift, I was managing patient care as usual. But I also started informally reminding nurses during the shift about the bundle steps. I brought it to my nurse manager and we developed a structured bundle checklist. So I was doing my daily job and also working on the improvement at the same time.”*—Nurse Manager, ICU, 3 years (P2)

##### Subtheme 2.3: Closing Behaviors—Sustaining Change Through Structure and Monitoring

Closing behaviors involved consolidating improvements through structured routines, protected meeting time, consistent monitoring, and deliberate modeling of new practices. Participants described these behaviors as essential to ensuring that changes were embedded rather than lost during busy periods.

“*I protect at least two hours per week for quality meetings and improvement planning. If I do not protect this time, it disappears. The balance is discipline—you must schedule both.”*—Nurse Manager, Medical Ward, 5 years (P1)

#### Theme 3: organizational agility as adaptive unit functioning

Participants described this unit-level agility as developing alongside sustained leadership practices and team experience, rather than as spontaneous team capacity.

##### Subtheme 3.1: Teams That Absorb the Unexpected

Participants described specific high-pressure episodes — surge events, equipment failures, critical deterioration — during which the team's agility was tested. In units where agility was strong, these events were managed without major adverse outcomes, with staff adapting in real time within pre-established frameworks.

“*During Hajj season, the hospital emergency department receives significantly increased patient volumes. We prepared an agile response plan—flexible staffing, clear escalation levels, converted a waiting area into additional assessment bays. When the surge came, the team responded according to the plan but also adapted in real time.”*—Nurse Manager, Emergency Department, 7 years (P3)

##### Subtheme 3.2: Delegation in adaptive unit functioning

Participants associated adaptive unit functioning with delegation, preparedness, and responsive decision-making. Participants described strategic delegation to charge nurses and senior staff as the strategy that freed nurse managers to manage both operations and improvement work simultaneously. Effective delegation was not simply task distribution but empowerment — building the capacity of others to make decisions independently.

“*I delegate clearly to charge nurses who are empowered to manage daily decisions. This frees me to focus on strategic management and improvement work. If operational data shows a problem, I temporarily shift attention there. The key is that both are always visible and never ignored.”*—Nurse Manager, Medical Ward, 12 years (P9)

##### Subtheme 3.3: Cultural and Religious Demands as Recurring Agility Requirements

A finding specific to the study hospital was the routine need to adapt to Ramadan, Hajj season, and family-centered care expectations. These predictable but demanding periods constituted a Saudi-specific organizational agility dimension that is absent from Western leadership frameworks and required purpose-built contingency planning.

“*During Ramadan, our obstetrics ward faces unique challenges — fasting staff, higher family visitation volumes, and sometimes changes in patient compliance with labour management. I implemented an agile staffing plan that accounted for fasting staff needs — adjusted break schedules, reduced heavy physical tasks during fasting hours, and prepared for extended visiting hours with clear protocols.”*—Nurse Manager, Obstetrics Ward, 8 years (P7)

#### Theme 4: innovation behaviors as problem-driven practices

Innovation in this sample consistently originated from a noticed problem rather than a planned strategy, reflecting the Innovative Behavior Inventory (IBI) framework enacted in everyday clinical management. Participants across all roles and experience levels described innovation as responsive and necessity-driven rather than proactively scheduled. Three subthemes emerged:

##### Subtheme 4.1: The Problem Comes First

Across all clinical settings, innovation was initiated by the recognition of a recurring gap in care quality, workflow efficiency, or patient experience. The identification of a problem created the impetus for generating ideas, seeking solutions, and building the case for change.

“*We had many patients complaining about pre-operative waiting times. They would wait in the ward for the surgery call for many hours without information. I kept managing daily ward operations but also worked with the OR team and bed management to design a new communication system — a board that shows expected surgery time updated every two hours. Patients were happier and the complaints decreased.”*—Nurse Manager, Surgical Ward, 4 years (P4)

##### Subtheme 4.2: Scale of Innovation Varies by Experience and Role

A clear gradient emerged across role levels: assistant nurse managers described bedside and documentation improvements; nurse managers with five or more years’ experience described ward-level quality projects; and clinical nurse directors described multi-departmental initiatives spanning a year or more. This gradient reflects the expanding sphere of influence and resource access associated with seniority.

“*I introduced a nurse-led palliative care consultation process within the oncology unit. Previously, palliative referrals were physician-initiated only and often delayed. Referral timeliness improved and patient comfort scores in their final weeks of care improved significantly.”*—Nurse Manager, Oncology Ward, 10 years (P5)

##### Subtheme 4.3: Working Through Staff Resistance

Resistance from experienced staff was the most commonly reported implementation barrier. The strategies participants used — explaining the rationale, co-modifying tools, and offering trial periods — directly reflect the ‘involving others’ and ‘overcoming obstacles’ dimensions of the IBI (21).

“*Some experienced nurses feel that new practices are criticism of their old ways. When we introduced new charting for pain assessment, one senior nurse said: ‘I know how to assess pain, I don*'t need a new form.’ I had to explain that it is not about doubting their skills but about having consistent documentation. Gradually she accepted it when she saw it helped during patient handover.”**—**Nurse Manager, ICU, 3 years (P2)

#### Theme 5: contextual challenges influencing leadership effectiveness

Four recurring constraints shaped what was possible for participants in enacting agile, ambidextrous, and innovative behaviors: physician hierarchy, staff emotional depletion, time scarcity, and the cultural dynamics of a multinational team serving patients whose expectations are shaped by Saudi cultural and religious norms. These constraints were not insurmountable, but they required deliberate and contextually sensitive leadership responses. Four subthemes were identified:

##### Subtheme 5.1: Physician Hierarchy

Participants described a persistent power asymmetry between nursing and medicine that could impede nursing-led initiatives. The most effective strategy for navigating this constraint was co-designing improvements with physician partners from the outset, framing proposals in terms of patient outcomes, and building evidence-based rationale before presenting to governance bodies.

“The medical hierarchy in Saudi healthcare can sometimes mean that nursing initiatives are not taken seriously without a physician champion. I address this by co-designing improvements with a physician partner from the beginning, building evidence-based rationale, and framing it in terms of patient outcomes rather than nursing preferences.”**— Nurse Manager, Oncology Ward, 10 years (P5)**

##### Subtheme 5.2: Staff Emotional State

Participants in high-acuity settings, particularly Oncology and ICU, described the emotional depletion of nursing staff as a significant barrier to engagement with improvement initiatives. Effective leaders in these contexts described reading the team's emotional temperature before introducing new demands and prioritizing psychological support over improvement agendas when needed.

“*When the team is grieving, introducing new practices or expecting engagement with improvement projects is very difficult. I have learned to read the emotional temperature of the team and adjust my leadership approach — sometimes what is needed is support and acknowledgment, not a quality meeting.”*—Nurse Manager, Oncology Ward, 4 years (P12)

##### Subtheme 5.3: Time Poverty Under Operational Pressure

Time scarcity was the most consistently reported barrier across all sites and experience levels. Less experienced managers described stopping improvement work during busy periods; more experienced managers described deliberately designing low-burden improvement activities that could run in parallel with operations, embedding measurement into existing workflows rather than adding separate processes.

“*The most persistent challenge is maintaining improvement momentum when operational demands surge. I have learned to design improvement projects with minimal operational burden—simple measurement tools, embedded checklists, brief team check-ins—so that improvement can continue even during busy periods.”*—Nurse Manager, Medical Ward, 12 years (P9)

##### Subtheme 5.4: Cultural Dynamics Within Teams and with Families

Participants described two intersecting cultural dynamics: managing expectations within a multinational nursing workforce with varying norms around hierarchy and authority, and navigating family expectations that sometimes conflicted with evidence-based practice. Both required culturally sensitive communication and, in the case of family expectations, structured staff preparation.

“*Some Saudi staff members expect a more hierarchical leadership style, and some more experienced nurses from any nationality can resist direction from a younger manager. I address this by leading with competence, being respectful of experience, and involving senior staff in decisions where their expertise is valuable.”*—Nurse Manager, ICU, 6 years (P18)

### Integrated quantitative and qualitative findings

Across the five themes, the qualitative findings gave substantive meaning to the highly interrelated quantitative profile. Participants described daily flexibility, environmental awareness, delegation, responsiveness, opening and closing behaviors, adaptive unit functioning, and problem-driven improvement as interconnected features of clinical management. These accounts help explain why the four survey measures may have converged so strongly in this setting without establishing that the constructs are empirically identical.

Participants’ accounts of opening and closing behaviors were embedded within the same practical narratives as delegation, operational responsiveness, and improvement work. The qualitative data therefore support interpretation of a broad agile leadership–organizational functioning profile rather than differentiation of construct-specific pathways. More experienced participants, and some with higher educational preparation, described these practices in more deliberate and system-oriented terms. This qualitative pattern was not tested quantitatively and remains hypothesis-generating.

Theme 5 added context that the survey measures did not capture. Physician hierarchy, staff emotional load, time constraints, and cultural dynamics shaped how managers described their leadership and improvement practices. These contextual findings show how agile leadership and organizational functioning were experienced within the institutional and cultural realities of Saudi clinical management.

Taken together, the integrated findings indicate that agile leadership, ambidextrous leadership, organizational agility, and innovation behaviors were experienced as highly interconnected aspects of nursing management. The quantitative findings provide descriptive and exploratory context for this interrelated profile, while the qualitative findings provide the primary interpretive insight into how these practices were experienced within the study setting. Contextual conditions, including physician hierarchy, workload and emotional demands, time constraints, and cultural dynamics, further shaped participants’ accounts of leadership and organizational functioning. Table 5 presents the joint display of the integrated quantitative and qualitative findings, while Figure 4 provides a visual summary of the integrated findings.

**Table 5:** Joint display of integrated quantitative and qualitative findings.

Quantitative Finding	Qualitative Theme	Supporting Participant Evidence	Integrated Interpretation
Near-unity covariation among the four measures (r = 0.977–.990)	Themes 1–4: Agile leadership, balancing behaviors, adaptive unit functioning, and problem-driven innovation	“I delegate clearly to charge nurses who are empowered to manage daily decisions.” (P9)	Participants described leadership, adaptation, and improvement as interconnected features of everyday management, providing context for the strong quantitative overlap.
Exploratory regression coefficients were unstable under severe multicollinearity	Themes 1 & 2: Multiple agile leadership practices enacted together	“Agile leadership means you don't manage from your office — you are on the floor, you see what is happening, and you respond.” (P22)	The qualitative findings do not support ranking one leadership construct over another. Participants described overlapping leadership practices.
Quantitative measures could not establish empirical separability	Themes 2–4: Interconnected leadership, unit functioning, and improvement practices	“When staff feel trusted, they act quickly without waiting.” (P19)	Trust, delegation, responsiveness, and improvement activity appeared together in participants’ accounts, consistent with a broader agile leadership–organizational functioning profile.
Same-source measurement and potential common-method influence limited construct-specific interpretation	Theme 5: Contextual conditions	“The most persistent challenge is maintaining improvement momentum when operational demands surge.” (P9)	Operational pressure and organizational context shaped participants’ perceptions and enactment of leadership and innovation, offering explanations not captured by the survey measures.
Qualitative finding only: experience-related developmental pattern	Themes 1 & 2: Reactive to anticipatory gradient	*“After twelve years… agility is not just reacting to crises. It is anticipating them.” (P10)*	More experienced participants described leadership in more anticipatory and system-oriented terms. This pattern was not quantitatively tested.
Contextual barriers not captured in quantitative model	Theme 5: Contextual challenges	*“The medical hierarchy can sometimes mean that nursing initiatives are not taken seriously without a physician champion.” (P5)*	Physician hierarchy, staff emotional load, and Saudi-specific cultural dynamics constrain leadership effectiveness and provide context for how agile leadership is experienced in this context.

AL, agile leadership; AMB, ambidextrous leadership; OA, organizational agility; IB, innovation behaviors; HAPI, hospital-acquired pressure injury.

**Figure 4:**
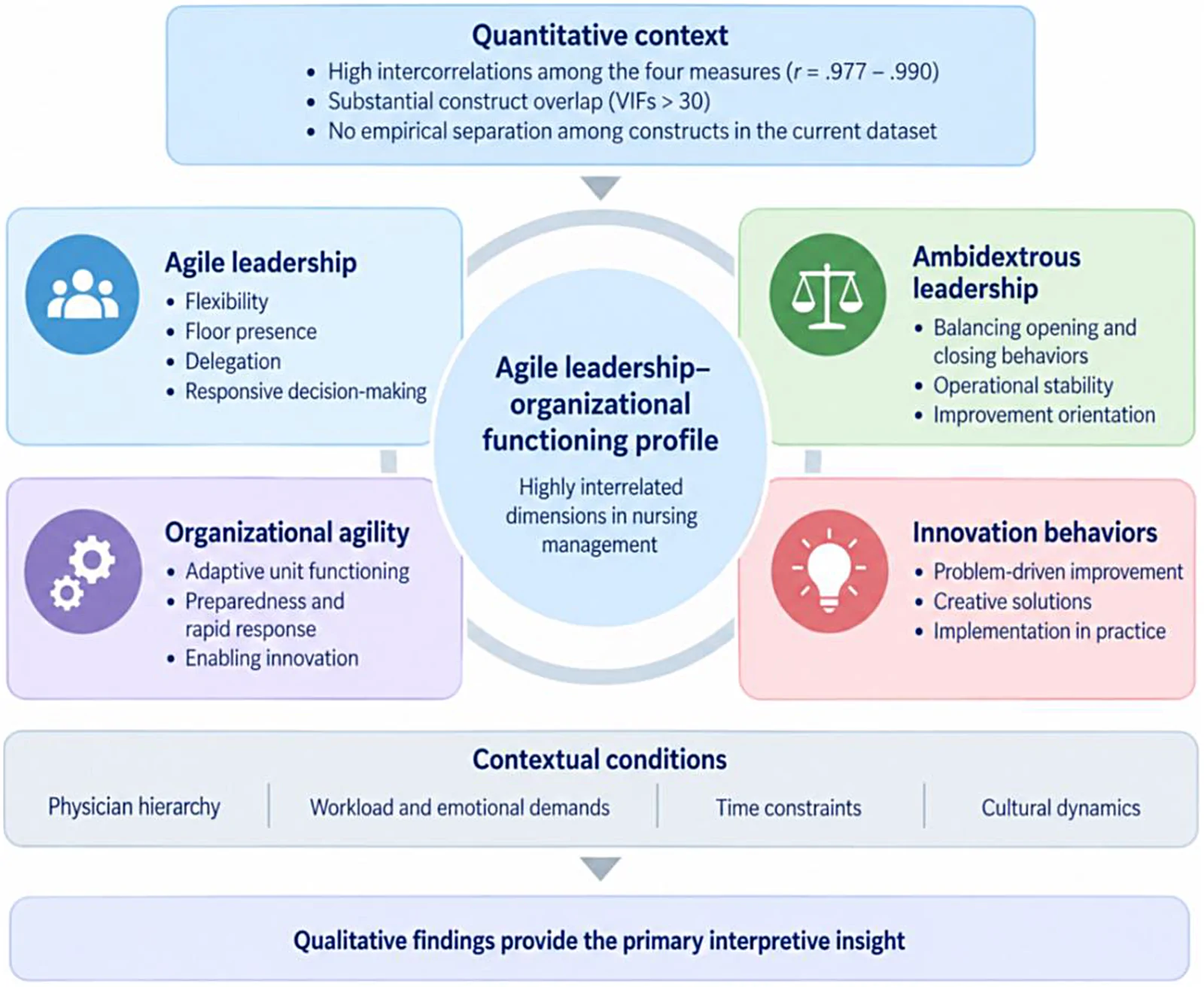
Integrated mixed-methods interpretation of the agile leadership–organizational functioning profile.

## Discussion

This study identified a highly interrelated agile leadership–organizational functioning profile among nurse managers. The quantitative measures moved almost in parallel, with correlations of 0.977–.990 and severe multicollinearity. These data therefore cannot establish distinct contributions for agile leadership, ambidextrous leadership, organizational agility, or innovation behaviors. The main contribution comes from the qualitative findings, which show how nurse managers experienced flexibility, floor presence, delegation, balancing opening and closing behaviors, adaptive unit functioning, problem-driven innovation, and contextual constraints in everyday clinical management.

### Descriptive-level findings

Nurse managers reported moderate levels of agile and ambidextrous leadership and high levels of organizational agility and innovation behaviors. These scores describe the study setting rather than distinct leadership contributions. Similar healthcare studies have reported associations among organizational agility, ambidextrous leadership, readiness for change, creativity, and innovative work behavior (5, 8, 14, 35). The present qualitative accounts add context by showing that managers described adjustment, delegation, anticipation, and improvement activity as part of routine clinical management.

Within the agile leadership subscales, self-awareness had the highest mean, followed closely by situational awareness and human relations. The differences were small and should not be interpreted as a developmental sequence. Özgenel and Yazıcı (12) conceptualized these as related dimensions of agile leadership, while Joiner (36) described leadership agility as involving the capacity to respond to complex and changing conditions. In the interviews, some more experienced managers described anticipating problems before escalation, whereas less experienced managers more often described responding to immediate changes. This remains a qualitative observation rather than evidence that experience produced higher agility.

For ambidextrous leadership, closing behaviors had a slightly higher mean than opening behaviors. The difference was small. Rosing et al. (7) describe opening behaviors as encouraging variation and experimentation and closing behaviors as supporting goal attainment, monitoring, and implementation. Healthcare studies have associated ambidextrous leadership with nurses’ creativity and innovative work behavior (8, 14), while Zacher and Rosing (28) showed that opening and closing behaviors may vary in relevance across conditions. In the present interviews, participants described both forms of behavior: opening practices through autonomy and idea sharing, and closing practices through monitoring, standards, and follow-up. The qualitative findings therefore contextualize the descriptive difference without assigning directional meaning to it.

### Innovation behaviors and organizational agility

Cross-sector research has also examined ambidextrous leadership, organizational agility, and innovation-related outcomes together (6). Because this evidence comes from a non-healthcare setting, it is used here only as comparative context rather than direct evidence for nursing management.

Among innovation behavior subscales, idea generation and idea search had the highest means, while overcoming obstacles had the lowest. Lukes and Stephan (21) distinguish idea generation and search from later implementation activities that require involving others and overcoming barriers. Nursing research similarly shows that innovation depends on organizational support, psychological empowerment, and implementation conditions rather than idea generation alone (20, 37, 38). In the interviews, staff resistance, physician hierarchy, and time pressure were frequently described when managers discussed implementation, providing contextual support for this descriptive pattern.

For organizational agility, quickness had the highest mean and flexibility the lowest. Sharifi and Zhang (18) distinguish quickness from flexibility as separate dimensions of organizational agility. In nursing settings, Hossny et al. (5) and Hussien et al. (19) reported relationships between organizational agility and readiness for change or staff outcomes. The present interviews described rapid response, contingency planning, and delegation during service demands, while changes requiring broader authority were described as more constrained. These accounts provide context for the observed scores without establishing why one agility dimension was higher than another.

### Construct overlap and agile leadership in practice

Although the exploratory regression models produced larger conditional coefficients for agile leadership, these coefficients cannot be interpreted as evidence that agile leadership was stronger or more distinct than ambidextrous leadership. Near-unity correlations and severe multicollinearity make the conditional coefficients unstable. The more defensible interpretation is that managers who reported higher agile leadership also tended to report higher ambidextrous leadership, organizational agility, and innovation behaviors.

Previous nursing and healthcare studies have reported relationships between agile or ambidextrous leadership and readiness for change, creativity, and innovative behavior (5, 8, 11, 14, 35). These studies support the relevance of the leadership practices examined here but do not resolve the measurement overlap in the present dataset. The contribution of this study therefore lies mainly in showing how Saudi nurse managers described agile leadership alongside organizational agility and innovation in everyday practice.

The qualitative findings add depth to this interpretation. Managers described monitoring the clinical environment, remaining visible, adjusting plans, delegating decisions, and anticipating problems. These accounts align with agile leadership literature (12, 23, 39), while also overlapping with organizational agility and innovation-related practices. The experience-related shift from reactive to anticipatory accounts warrants prospective examination rather than a developmental conclusion.

### Ambidextrous leadership within the broader agile profile

The ambidextrous leadership measure was almost perfectly correlated with agile leadership and strongly correlated with organizational agility and innovation behaviors. Its coefficient changed when modeled alongside the overlapping agile leadership measure, illustrating coefficient instability rather than a distinct association. Rosing et al. (7) conceptualized ambidextrous leadership as the flexible use of opening and closing behaviors, and Zacher and Rosing (28) found that these behaviors were related to team innovation under different conditions. In healthcare, El-Gazar et al. (8) reported associations between ambidextrous leadership, psychological safety, and nurses’ creativity, while El-Sayed et al. (14) reported an association with innovative work behavior among critical care nurses. These studies support the relevance of ambidextrous behaviors but do not justify interpreting the unstable coefficient in this highly collinear dataset as a unique contribution.

A notable qualitative finding was that many participants were unfamiliar with the formal term ambidextrous leadership even when they described practices resembling opening and closing behaviors. This distinction between conceptual familiarity and practical enactment may be useful for leadership education. The pattern is consistent with the behavioral framing of ambidextrous leadership proposed by Rosing et al. (7) and with nursing evidence linking such behaviors with creativity and innovative work behavior (8, 14). The present interviews extend this literature by showing how Saudi nurse managers described experimentation, autonomy, monitoring, and implementation discipline within routine clinical management. Future studies with larger samples and adequate measurement models are needed to determine whether ambidextrous leadership can be empirically distinguished from agile leadership in this population.

### Organizational agility and innovation within everyday unit functioning

Organizational agility and innovation behaviors were also near-unity correlated with the leadership measures. The present study therefore does not interpret organizational agility as an intervening mechanism between leadership and innovation. Prior nursing studies have reported relationships between organizational agility and readiness for change or staff well-being (5, 19), while nursing innovation studies have linked leadership and organizational conditions with innovative behavior (14, 20, 37, 38). These studies provide empirical context for examining the concepts together, but the present cross-sectional data cannot determine their temporal ordering.

Theme 3 described organizational agility through delegation, preparedness, rapid response, and advance planning for changing service demands. Theme 4 portrayed innovation as problem-driven practice emerging from clinical and workflow needs. These accounts are consistent with nursing studies in which organizational agility was related to readiness for change (5, 19) and leadership or organizational conditions were associated with innovative behavior (37, 38). In the present interviews, adaptation and improvement often appeared within the same managerial accounts. Saudi-specific conditions, including Ramadan staffing patterns, Hajj-related demand, and family-centred care expectations, added context to how these practices were described.

Periods of high workload and limited time constrained improvement activity, whereas delegation, flexibility, and organizational support were described as conditions that helped managers continue operational and improvement work. This interpretation is consistent with nursing innovation research emphasizing organizational support, psychological empowerment, collaboration, and implementation conditions (20, 39). The interviews add context by showing how managers described these demands in a Saudi tertiary hospital. These findings do not establish temporal ordering or directional pathways.

Accordingly, the present findings do not support decomposition of the observed associations into direct and indirect pathways. Such decomposition would imply empirical separation among constructs that could not be demonstrated in this dataset.

Future research can test whether agile leadership, ambidextrous leadership, organizational agility, and innovation behaviors represent distinct latent constructs or dimensions of a broader agile leadership–organizational functioning profile. This requires larger multisite samples, confirmatory measurement models, longitudinal designs, and multi-source data (28, 40).

### Empirical overlap Among leadership and organizational constructs

Despite their theoretical distinction, agile and ambidextrous leadership were almost perfectly correlated in this sample (r = 0.988), and both were also highly correlated with organizational agility and innovation behaviors. Shared behavioral content, same-source self-report, response tendencies, and very high internal consistency may have contributed to this overlap. The present data therefore cannot establish discriminant validity among the constructs, which should be examined in larger samples using confirmatory measurement models.

### Integrated interpretation of mixed-methods findings

The qualitative findings are the principal contribution of this study because they show how agile and ambidextrous leadership, organizational agility, and innovation were experienced in clinical practice. Managers described responsiveness, delegation, opening and closing behaviors, unit functioning, and problem-driven innovation as intertwined activities. This pattern contextualizes the quantitative overlap without assuming distinct pathways. Theme 5 further demonstrates why context matters. Physician hierarchy, staff emotional depletion, time scarcity, and cultural complexity shaped managers’ accounts of leadership and improvement. These findings add a Saudi healthcare perspective not captured by the survey scores alone (5, 19, 41).

A developmental pattern emerged in the qualitative data, with more experienced managers describing leadership in more anticipatory and system-oriented terms. Because managerial experience was not tested as a predictor, moderator, or longitudinal exposure in the quantitative models, this finding should be interpreted as qualitative and hypothesis-generating rather than as evidence of developmental change over time.

Accordingly, the study is best understood as an exploratory mixed-methods account of a highly interrelated agile leadership–organizational functioning profile. The quantitative data describe the profile and its measurement limitations, while the qualitative data provide the primary insight into how leadership, organizational agility, innovation, and contextual constraints were experienced.

### Alternative Explanations

The very high correlations among study constructs also raise the possibility that factors outside the proposed model contributed to the observed pattern of results. A shared, unit-level organizational culture or leadership climate — rather than individual leaders’ agile or ambidextrous behaviors specifically — could inflate correlations among all four constructs if respondents’ ratings partly reflect a general impression of “how good things are around here” rather than construct-specific judgments (42). Psychological safety, which was raised repeatedly in the qualitative interviews as a precondition for both opening behaviors and innovation, is one plausible common-cause candidate that was not directly measured. Social desirability is another: because all instruments were completed by the same nurse managers being asked to describe their own leadership and their unit's functioning, motivated positive self-presentation could inflate scores on all scales simultaneously. Finally, institution-wide policies and initiatives connected to Vision 2030 — digitalization mandates, quality-improvement targets, accreditation cycles — apply uniformly across the study hospital and could produce correlated variation in agility and innovation ratings independent of any specific leader's behavior. The present cross-sectional, single-source design cannot separate these alternative explanations from the hypothesized leadership relationships, and this is an important boundary condition on the study's conclusions.

Although agile and ambidextrous leadership are theoretically distinct, their near-unity correlation in this sample indicates limited empirical separation. Both instruments capture behaviors related to responsiveness, flexibility, empowerment, adaptation, and managing change. Nurse managers may therefore have responded to both measures through a broader self-perception of agile leadership competence. Same-source measurement, common response tendencies, and the very high internal consistency of several scales may have further increased empirical overlap. The present findings cannot establish whether the constructs represent distinct latent dimensions in this population.

### Strengths and Limitations

The mixed-methods design was a strength because the qualitative phase provided detailed contextual explanation of the quantitative patterns. The study also achieved a high census response among eligible nurse managers (44 of 50 eligible nurse managers, 88%) and included participants from diverse clinical specialties and professional backgrounds.

Several limitations are central to interpretation. First, the quantitative phase included only 44 nurse managers from one hospital. Although this represented 88% of the eligible population, the sample was too small for stable confirmatory factor analysis or structural equation modeling. The study therefore cannot establish discriminant validity among the four theoretical constructs. Second, correlations approached unity and VIF values were markedly elevated, indicating severe multicollinearity and substantial empirical overlap. Regression coefficients are therefore unstable descriptive outputs rather than estimates of unique construct-specific contributions. Third, all quantitative measures were self-reported by the same respondents at the same time, creating substantial common-method concern. Harman's first factor accounted for 58.16% of variance, although this diagnostic cannot quantify common-method bias. Fourth, very high alpha coefficients may partly reflect item redundancy. Fifth, the cross-sectional design precludes temporal or directional inference and does not support mediation or indirect-pathway claims. Finally, qualitative participants came from the same respondent pool, so their accounts provide contextual explanation rather than independent validation of the quantitative findings.

### Implications

#### Implications for Nursing Practice and Healthcare Management

− The findings identify leadership practices that warrant further evaluation in nursing management, including structured delegation, adaptive decision-making, opportunities for improvement work, and support for early-career nurse managers. The qualitative findings also highlight psychological safety, interprofessional collaboration, managerial discretion, and responsiveness to changing clinical demands as relevant contextual considerations (43).
− Healthcare organizations may consider these areas when reviewing leadership development and mentorship initiatives. Given the exploratory quantitative findings and the single-site, cross-sectional design, their usefulness should be evaluated prospectively before specific organizational standards, performance requirements, or structured interventions are recommended.

#### Implications for Nursing Education and Leadership Development

− Leadership development programs may use agile and ambidextrous leadership concepts as frameworks for examining agile leadership practices. Educational activities could address delegation, adaptive decision-making, opening and closing leadership behaviors, and balancing operational requirements with improvement activities.
− Simulation and case-based learning may provide opportunities to examine leadership responses to situations relevant to the Saudi healthcare context, including multicultural team dynamics, periods of increased service demand, interprofessional collaboration, and changing clinical priorities. Mentorship for early-career nurse managers may also provide a relevant focus for future leadership-development initiatives.

#### Implications for Policy

− The findings may inform discussion about agile leadership, nursing participation, managerial discretion, and organizational flexibility within Saudi healthcare workforce-development initiatives aligned with Vision 2030. The qualitative findings indicate that nurse managers perceived interprofessional participation, organizational support, and involvement in improvement activities as relevant to their leadership practice.
− Cross-sector evidence suggests that collaborative governance is associated with institutional processes, participation, role clarity, and operational mechanisms (44, 45). Although this evidence is not specific to nursing or healthcare, it provides an organizational perspective relevant to examining nursing participation in clinical decision-making and quality-improvement structures. Further healthcare-specific evidence is needed before translating these findings into organizational or national policy recommendations.

#### Implications for Future Research

− Future studies should use larger multisite samples to examine whether the observed associations are reproduced across healthcare settings. Larger samples would permit confirmatory factor analysis and assessment of convergent and discriminant validity to determine whether agile leadership, ambidextrous leadership, organizational agility, and innovation behaviors represent empirically distinct constructs. Research could also examine whether these constructs load onto separate latent factors or whether a higher-order agile leadership–organizational functioning factor better represents their empirical relationships.
− Longitudinal designs are needed to examine temporal relationships among leadership, organizational agility, and innovation behaviors. The experience-related pattern identified qualitatively should be tested quantitatively by examining managerial experience as a predictor or moderator rather than assuming developmental progression.
− Multi-source designs could incorporate staff ratings of leadership, organizational indicators, quality-improvement measures, and relevant patient outcomes to reduce reliance on manager self-report. Once the measurement structure and relationships among the constructs are established, prospective intervention studies could evaluate specific leadership-development approaches.
− Further qualitative and mixed-methods research could examine contextual influences identified by participants, including multicultural workforce dynamics, periods of increased service demand, interprofessional relationships, and organizational support for improvement activities. The relationship of digital and e-leadership practices with agile and ambidextrous leadership also warrants investigation in nursing management.

## Conclusion

Agile leadership, ambidextrous leadership, organizational agility, and innovation behaviors represent important dimensions of contemporary nursing management, particularly in healthcare environments that require responsiveness, flexibility, coordinated decision-making, and continuous improvement. Understanding how these dimensions are experienced together can inform the development of nursing leadership and organizational practices that support managers in responding to changing clinical and operational demands.

In this study, the four quantitative measures formed a highly interrelated agile leadership–organizational functioning profile. Their near-unity correlations and substantial empirical overlap did not permit reliable differentiation of construct-specific relationships. The quantitative findings therefore provide descriptive and exploratory context rather than evidence of distinct contributions of individual constructs.

The qualitative findings provide the main contribution of the study by showing how this profile was expressed in everyday nursing management. Nurse managers described flexibility, floor presence, delegation, opening and closing leadership behaviors, responsiveness to operational demands, and problem-driven innovation. Their accounts also showed how physician hierarchy, workload, staff emotional demands, time constraints, and cultural dynamics shaped the enactment of these practices within the Saudi healthcare context.

Together, the findings highlight agile leadership and organizational functioning as interconnected areas of relevance to nursing management while also identifying an important measurement question regarding the distinctiveness of the four constructs. Future multisite, longitudinal, and multi-source studies with larger samples and confirmatory measurement models are needed to clarify their empirical boundaries and examine how these leadership and organizational practices relate to nursing management and innovation across healthcare settings.

## Data Availability

The original contributions presented in the study are included in the article/Supplementary Material, further inquiries can be directed to the corresponding author.
