# Supplementary material for: Exploring agile leadership, ambidextrous leadership, organizational agility, and innovation behaviors among nurse managers: an explanatory mixed-methods study
Source: Front Health Serv. 2026 Oct 1;6:1950319. doi: 10.3389/frhs.2026.1950319 (PMC13668840; doi:10.3389/frhs.2026.1950319)
Supplement: Supplementary file 1 [file Table1.docx]

Supplementary Table 1

*Pearson Correlation Matrix Among Main Study Variables*

| **Variable** | **AL** | **AMB** | **OA** | **IB** |
| --- | --- | --- | --- | --- |
| 1. Agile Leadership (AL) |  |  |  |  |
| 2. Ambidextrous Leadership (AMB) | .988*** |  |  |  |
| 3. Organizational Agility (OA) | .984*** | .977*** |  |  |
| 4. Innovation Behaviors (IB) | .989*** | .982*** | .990*** |  |

***Note.****. *** p < .001.*

Supplementary Table 2

*Demographic and Professional Characteristics of Qualitative Participants (N = 32)*

| **P#** | **Position** | **Work Unit** | **Exp. (yrs)** | **Nationality** |
| --- | --- | --- | --- | --- |
| P1 | Nurse Manager | Medical Ward | 5 | Saudi |
| P2 | Nurse Manager | ICU | 3 | Filipino |
| P3 | Nurse Manager | Emergency Dept. | 7 | Saudi |
| P4 | Nurse Manager | Surgical Ward | 4 | Indian |
| P5 | Nurse Manager | Oncology Ward | 10 | Saudi |
| P6 | Nurse Manager | Pediatric Ward | 6 | Jordanian |
| P7 | Nurse Manager | Obstetrics Ward | 8 | Saudi |
| P8 | Nurse Manager | ICU | 2 | Egyptian |
| P9 | Nurse Manager | Medical Ward | 12 | Saudi |
| P10 | Nurse Manager | Surgical Ward | 5 | Filipino |
| P11 | Nurse Manager | ICU | 9 | Saudi |
| P12 | Nurse Manager | Oncology Ward | 4 | Indian |
| P13 | Nurse Manager | Pediatric Ward | 6 | Saudi |
| P14 | Nurse Manager | Emergency Dept. | 3 | Jordanian |
| P15 | Nurse Manager | Medical Ward | 7 | Saudi |
| P16 | Nurse Manager | Surgical Ward | 2 | Filipino |
| P17 | Nurse Manager | Obstetrics Ward | 11 | Saudi |
| P18 | Nurse Manager | ICU | 6 | Indian |
| P19 | Nurse Manager | Medical Ward | 14 | Saudi |
| P20 | Nurse Manager | Surgical Ward | 4 | Jordanian |
| P21 | Nurse Manager | Emergency Dept. | 5 | Saudi |
| P22 | Nurse Manager | Pediatric Ward | 6 | Filipino |
| P23 | Nurse Manager | Oncology Ward | 8 | Saudi |
| P24 | Nurse Manager | Medical Ward | 3 | Indian |
| P25 | Nurse Manager | ICU | 9 | Saudi |
| P26 | Nurse Manager | Surgical Ward | 7 | Jordanian |
| P27 | Nurse Manager | Pediatric Ward | 5 | Saudi |
| P28 | Nurse Manager | Medical Ward | 4 | Filipino |
| P29 | Nurse Manager | Obstetrics Ward | 13 | Saudi |
| P30 | Nurse Manager | ICU | 5 | Indian |
| P31 | Nurse Manager | Surgical Ward | 6 | Saudi |
| P32 | Nurse Manager | Emergency Dept. | 3 | South African |

*Supplementary Table 3*

*Theme and Subtheme Supported by Quotations*

| **Theme** | **Sub-themes** | **Illustrative Quotation** |
| --- | --- | --- |
| **Theme 1 Agile Leadership as Flexibility and Human-Centered Responsiveness** | 1.1 Real-time adjustment to clinical demands | *“I cannot stay fixed in one plan. I observe the situation in the morning, I adjust my team assignment, and by afternoon I may change again.” (P1, NM, Medical Ward)* |
|  | 1.2 Presence and floor visibility | *“Agile leadership means you don’t manage from your office — you are on the floor, you see what is happening, and you respond.” (P22, NM, Pediatric Ward)* |
|  | 1.3 From reactive to anticipatory: experience gradient | *“Agility is not just reacting to crises. It is anticipating them and positioning your team to respond before the crisis fully develops.” (P9, NM, Medical Ward, 12 yrs)* |
|  | 1.4 The human dimension: responding to people, not just situations | *“We shouldn’t think only about their work outcome… we should think about their situation and their life.” (P7, NM, Obstetrics Ward)* |
| **Theme 2 Ambidextrous Leadership as Balancing Opening and Closing Behaviors** | 2.1 Unfamiliarity with the concept; intuitive practice | *“This is the first time I heard this term. Maybe in leadership it means you handle today’s work and also plan for future improvement? I honestly do not know the proper academic definition.” (P4, NM, Surgical Ward)* |
|  | 2.2 Opening behaviors: creating space for new ideas | *“I was doing my daily job and also working on the improvement at the same time.” (P2, ANM, ICU)* |
|  | 2.3 Closing behaviors: sustaining change through monitoring | *“I protect at least two hours per week for quality meetings and improvement planning. If I do not protect this time, it disappears.” (P1, NM, Medical Ward)* |
| **Theme 3 Organizational Agility as Adaptive Unit Functioning** | 3.1 Unit-level absorption of the unexpected | *“When the surge came, the team responded according to the plan but also adapted in real time. We had no major adverse events.” (P3, NM, Emergency Dept., 7 yrs)* |
|  | 3.2 Delegation as the structural engine of agility | *“I delegate clearly to charge nurses who are empowered to manage daily decisions. This frees me to focus on strategic management and improvement work.” (P9, NM, Medical Ward, 12 yrs)* |
|  | 3.3 Cultural and religious demands as recurring agility requirements | *“During Ramadan, I implemented an agile staffing plan that accounted for fasting staff needs — adjusted break schedules, reduced heavy physical tasks during fasting hours.” (P7, NM, Obstetrics, 8 yrs)* |
| **Theme 4 Innovation Behaviors as Problem-Driven Practices** | 4.1 Bedside observation triggers improvement | *“I noticed that some patients were having hypoglycaemic episodes in the evening because monitoring frequency was not adjusted for their mealtimes. I raised this with the medical team.” (P28, NM, Medical Ward)* |
|  | 4.2 Scale of innovation varies by seniority and role | *“Over the past two years, I led a systematic effort to reduce our ward’s hospital-acquired pressure injury rate from 3.2% to 0.8%.” (P9, NM, Medical Ward, 12 yrs)* |
|  | 4.3 Working through staff resistance | *“Giving people the chance to experience the change, rather than just being told about it, makes a difference.” (P10, NM, Surgical Ward)* |
| **Theme 5 Contextual Challenges Influencing Leadership Effectiveness** | 5.1 Physician hierarchy as a structural barrier | *“The medical hierarchy can sometimes mean that nursing initiatives are not taken seriously without a physician champion.” (P5, CND, Oncology, 10 yrs)* |
|  | 5.2 Staff emotional state as prerequisite for innovation | *“When the team is grieving, introducing new practices or expecting engagement with improvement projects is very difficult.” (P12, NM, Oncology)* |
|  | 5.3 Time poverty under operational pressure | *“Managing the daily emergency is already more than enough. Maybe with more experience and training I will be better at balancing both.” (P32, NM, Emergency Dept.)* |
|  | 5.4 Cultural dynamics within teams and with families | *“Some Saudi staff members expect a more hierarchical leadership style… I address this by leading with competence and involving senior staff in decisions where their expertise is valuable.” (P18, NM, ICU)* |
